# Edifying the Focal Factors Influencing Mesenchymal Stem Cells by the Microenvironment of Intervertebral Disc Degeneration in Low Back Pain

**DOI:** 10.1155/2022/6235400

**Published:** 2022-03-27

**Authors:** Maite Esquijarosa Hechavarria, Seidu A. Richard

**Affiliations:** ^1^Department of Anesthesia and Critical Care, School of Medicine, University of Health and Allied Sciences, Ho, Ghana; ^2^Department of Medicine, Princefield University, P.O. Box MA 128, Ho, Ghana

## Abstract

Intervertebral disc degeneration (IVDD) is one of the main triggers of low back pain, which is most often associated with patient morbidity and high medical costs. IVDD triggers a wide range of pathologies and clinical syndromes like paresthesia, weakness of extremities, and intermittent/chronic back pain. Mesenchymal stem cells (MSCs) have demonstrated to possess immunomodulatory functions as well as the capability of differentiating into chondrocytes under appropriate microenvironment conditions, which makes them potentially epitome for intervertebral disc (IVD) regeneration. The IVD microenvironment is composed by niche of cells, and their chemical and physical milieus have been exhibited to have robust influence on MSC behavior as well as differentiation. Nevertheless, the contribution of MSCs to the IVD milieu conditions in healthy as well as degeneration situations is still a matter of debate. It is still not clear which factors, if any, are essential for effective and efficient MSC survival, proliferation, and differentiation. IVD microenvironment clues such as nucleopulpocytes, potential of hydrogen (pH), osmotic changes, glucose, hypoxia, apoptosis, pyroptosis, and hydrogels are capable of influencing the MSCs aimed for the treatment of IVDD. Therefore, clinical usage of MSCs ought to take into consideration these microenvironment clues during treatment. Alteration in these factors could function as prognostic indicators during the treatment of patients with IVDD using MSCs. Thus, standardized valves for these microenvironment clues are warranted.

## 1. Introduction

Intervertebral disc degeneration (IVDD) is one of the main triggers of low back pain, which is most often associated with patient morbidity and high medical costs [1, 2]. IVDD triggers a wide range of pathologies and clinical syndromes such as paresthesia, weakness of extremities, and intermittent/chronic back pain [2]. Cell senescence of intervertebral discs (IVDs), which is an indication of degeneration was detected as early as age of eleven [3–5]. Cell senescence is one of the most prominent pathways via which IVD cells decrease, resulting in IVDD [3]. Also, histological proof of reduced blood supply to the vertebral endplates was observed in the second decade of life and more predominant in advanced ages [3, 6].

The most current promising treatment modality for IVDD is cell-based therapy because it has demonstrated to possess disc regenerative potential with very minimally invasions compared to surgery [7, 8]. This therapeutic modality targets disc inflammation via blockade of anomalous cytokine generation, disc rehydration, and height restoration via the stimulation of matrix anabolism, repopulating and restoring the disc native cells [7]. Disc cells, notochordal cells, and stem cells are the key types of cell therapy for the treatment of IVD regeneration [7, 9]. Bach et al. demonstrated that healthy notochordal cell-derived NP tissue matrix was capable of harnessing the notochordal cells regenerative potential and implicated it for biological IVD repair [10].

Mesenchymal stem cells (MSCs) have demonstrated to possess immunomodulatory functions as well as the capability of differentiating into chondrocytes, which forms cartilage, making these cells potentially epitome for intervertebral disc (IVD) regeneration under appropriate microenvironment conditions [7, 11, 12]. Studies demonstrated that, MSCs obtained from bone marrow as well as adipose tissue were capable of differentiating into nucleus pulposus (NP)-like phenotype such as collagen fibrils and proteoglycan [11, 13]. Also, coculture trials of MSCs with NPCs revealed proliferation of the NPCs as well as differentiation of the MSCs into chondrogenic cells [7, 14]. Grafted MSCs were capable of restoring IVDD back to the normal disc milieu via the stimulation of the generation of extracellular matrix proteins such as aggrecan, proteoglycan, and collagen type-II, which constitute NPCs [7, 11, 15, 16].

The niche of the IVD milieu, which is often composed of the niche cells itself as well as their chemical and physical milieu, has been demonstrated to have a robust influence on MSC behavior as well as differentiation [17]. Nevertheless, the influence of MSCs by the IVD milieu conditions in healthy as well as IVDD situations is still a matter of debate. It is still not clear, which factors, if any, are essential for effective and efficient MSC survival, proliferation, and differentiation. Intradiscal injection of MSCs is a promising minimally invasive method of treating IVDD [18]. This mode of administration of MSCs determines therapeutic efficacy at different stages of IVDD [18].

This review thus edifies the immunomodulatory roles of MSCs at the microenvironment of IVDD. The “Boolean logic” was used to search for an article on the subject matter. Most of the articles were indexed in PubMed and/or PMC with strict inclusion criteria being the immune players that are influenced by MSCs during transplantation in human or animal models of IVDD. The search teams were a combination of MSCs and/or the immunome players, which constitute headings in the paper.

## 2. Anatomy of the Intervertebral Disc and Degeneration

The IVD is composed of three interrelated connective tissue components such as the NP, annulus fibrosus (AF), and hyaline cartilaginous endplates (CEPs) [1, 19]. The NP is specifically made up of hydrated tissue composed of copious proteoglycans and relatively low collagen content and located within the core of the disc for compression resistance and torsion, respectively. A study revealed that the glycosaminoglycan to collagen ratio was about 27:1, while the ratio of CEPs in contrast was about 2:1 in the NP of young adults [19]. It was also established that hydration of the NP triggered a swelling pressure that was opposed by the circumferential AF, which encloses the NP compartment and constitutes a sprouting junction with adjacent vertebrae [19].

The AF consists of concentric, collagenous lamellae intertwined with proteoglycan-rich septae [19, 20]. Histologically, the AF is separated into two zones including the inner collagen type-II as well as proteoglycan-rich zone and the outer collagen type-I-rich zone by a categorized modification [19, 20]. Morphologically dissimilar annulocytes have been observed at the cellular level [19, 20]. These cell categories include cells with widespread sinuous processes situated within the inner AF, cells with wide and branching processes precisely located in the interlamellar space of the outer AF, and interlocked cord-like cells situated at the outer zones [19, 20]. The hyaline CEPs are located at the boundary between the disc tissue and vertebral bodies. The vertebral bodies are porous and allow diffusion of molecules delivered via the extra-discal capillary beds [19, 21].

The NP, AF, and CEPs units as well as neighboring vertebral bodies form a polyaxial diarthrodial joint [19, 22]. This composition permits integrated movements and can deform along six degrees of freedom [19, 22]. The IVD is an avascular tissue and sections of the NP are found 8 mm from the closest blood supply [3, 6]. Nevertheless, the IVDs are fed via two distinct capillary plexuses. The first feeds the outer AF while the second originates from the vertebral bodies and feeds the bone-cartilage junction [3, 6]. Nutrition such as glucose, oxygen, and macromolecules are driven by a diffusion gradient through spinal movements. Anaerobic metabolism is often observed within the NP because the cells within it have low oxygen (O_2_) tension due to the limited blood supply [3]. Furthermore, the milieu of the NP has higher levels of lactic acid and a lower pH than other sections of the disk, which influence cell function negatively [3].

The degenerative process of the IVD often starts with disc herniation, which is depicted with protrusion of the NP as a result of wear and tear, repetitive mechanical overloading, and trauma/injury into the spinal canal [19, 23]. In severe situations, the endplate may rupture and protrude into the vertebral marrow [19, 23]. The extrusion into the spinal canal or the vertebral marrow compresses on vertebral marrow and triggers immune cascades [19, 23]. Subsequently, the central spinal canal or spinal foramen via which spinal nerves exit the column becomes narrower (spinal stenosis) as a result of bony overgrowth [19, 24].

Furthermore, displacement of a vertebra over another, which is often referred to as degenerative spondylolisthesis, occurs [19, 25]. Moreover, the spinal curvature digresses aberrantly as the discs collapse and facet joints degenerate in an asymmetric manner, which is also referred to as degenerative scoliosis[19, 26]. Finally, discogenic back pain, which is triggered by the ingrowth of nociceptive nerve fibers along the outer AF and annular tears, occurs, and the patients at this point need urgent attention because of pain [19, 27].

## 3. Nucleopulpocytes (NPCys)

**Figure 1:**
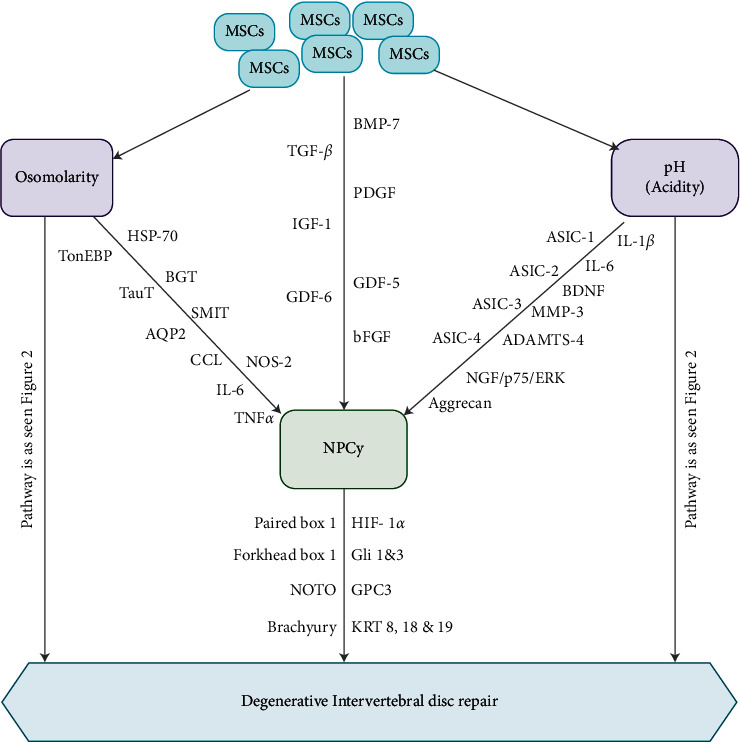
Illustration showing the pathway via which MSCs influence NPCy at the microenvironment leading to repair of IVDD. Factors such as TGF-*β*, GDF-5 and 6, BMP-7, PDGF, IGF-1, and *β*FGF are responsible for the differentiation of MSCs towards NPC-like cells. Also, acidity and osmolarity via diverse signaling pathways are key factors influencing the MSCs/NPC phenotype.

Nucleopulpocytes (NPCys) are chondrocyte-like round cells usually located within the NP [4, 28]. These cells are depicted with precise markers such as hypoxia-inducible factor (HIF) 1*α*, glioma-associated oncogene (Gli) 1 and 3, glypican 3 (GPC3), paired box 1 and forkhead box 1, NOTO, and brachyury as well as keratin (KRT) 8, 18, and 19 (Figure 1) [4, 29, 30]. MSCs are capable of differentiating into NPCy-like phenotypes. MSCs sustainability, as well as differentiation into a NPCy-like phenotype, was augmented by growth factors like transforming growth factor beta (TGF-*β*), platelet-derived growth factor (PDGF), insulin-like growth factor 1 (IGF-1), growth differentiation factor (GDF)-5, and GDF-6 as well as basic fibroblast-like growth factor (bFGF) (Figure 1) [4].

These molecules above are typically secreted by IVD resident cells. Nevertheless, cell density reduced and nutrient diffusion hampered; the extracellular membrane/matrix (ECM) turns out to be disrupted and their anabolic influence becomes nearly totally diminished [31]. Therefore, presubjecting MSCs *in vitro* to these growth factors before grafting or implanting them in MSC-loaded bio-frameworks could preserve their biological activity as well as enhance cell proliferation, differentiation, and synthetic potential [4, 31]. It is established that the potential of hydrogen (pH) changes are often detected by IVD cells via acid-sensing ion channels (ASICs), which regulate Ca^2+^ transmembrane influx upon instabilities of extracellular H^+^ levels [4].

It was observed that human IVD cells were capable of secreting ASIC isoforms such as ASIC-1, ASIC-2, and ASIC-3 as well as ASIC-4. ASIC-1 stimulation resulted in augmented Ca^2+^ cellular influx, leading to apoptosis of CEP chondrocytes (Figure 1) [4, 32]. Furthermore, ASIC-3 was implicated in NPCy survival under acidic as well as hyperosmolar microenvironments via the nerve growth factor (NGF)/p75/extracellular signal-related kinases (ERK) pathway (Figure 1) [4, 33]. A study revealed that ASIC secreted by NPCy and AF cells is mostly elevated in IVDD [34]. Gilbert et al. demonstrated that human NPCy viability was high, while proliferation was abrogated in an *in vitro* pH of 6.8 [35].

It was also noted that cells undergo necrosis at lower pH and express substantial quantities of proinflammatory cytokines such as IL-1*β* and ΙL-6, neurotrophic pain-related factors such as NGF, and brain-derived neurotrophic factor (BDNF) (Figure 1) [4]. Nevertheless, highly acidic culture milieu was associated with a reduction in aggrecan levels together with elevation of matrix metalloproteinases (MMP)-3, a disintegrin and metalloproteinase with thrombospondin motifs (ADAMTS)-4, and ASIC-3, whilst ASIC-1 and ASIC-2 quantities remained unchanged. MSCs were capable of expressing IGF-1 and bone morphogenetic protein (BMP)-7, which protected NPCy against apoptosis (Figure 1) [4, 36].

NPCys are capable of detecting variations of osmolarity by stimulating the secretion of tonicity enhancer-binding protein (TonEBP) [4, 37, 38]. In a hypertonic milieu, the TonEBP, in turn, binds to the tonicity-responsive enhancer element (TonE) motif, leading to the elevation of genes like heat shock protein (HSP)-70, betaine/*γ*-aminobutyric acid transporter (BGT), sodium myo-inositol transporter (SMIT), taurine transporter (TauT), and aquaporin-2(AQP2), which are essential for the regulation of intracellular osmotic stress (Figure 1) [4, 37, 38]. TonEBP has been associated with the upregulation of proinflammatory cytokines stress such as C-C motif chemokine ligand (CCL), nitric oxide synthase 2 (NOS-2), interleukin (IL)-6, and TNF*α* under hypertonic (Figure 1) [4, 39].

It is affirmed that the hypertonic culture microenvironment decreased bone marrow-derived mesenchymal stem cell (BM-MSC) proliferation, anabolic marker secretion, and chondrogenic differentiation [4, 17]. Furthermore, adipose-derived mesenchymal stem cell (AD-MSC) viability and proliferation in addition to aggrecan and collagen type-I secretion were inhibited [4, 40]. Li et al. demonstrated that introduction of nucleus pulposus mesenchymal stem cells (NP-MSCs) to osmotic pressures that mimicked the healthy IVD environment led to a reduction in cell proliferation and chondrogenic differentiation via stimulation of the ERK pathway, while comparatively, the hypoosmotic microenvironment of mild IVDD revealed an upsurge of NP-MSC proliferation as well as chondrogenic potential [41].

## 4. Potential of Hydrogen (pH)

**Figure 2:**
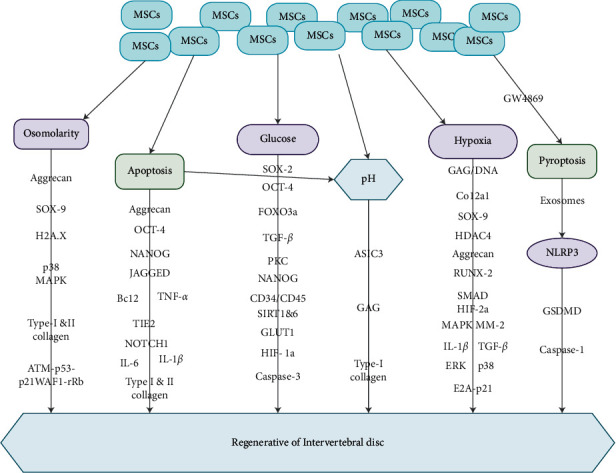
Illustration showing the various microenvironment clues and the pathways via which MSCs influence the repair of IVDD. MSCs influence osmolarity, apoptosis, glucose, pH, hypoxia, and pyroptosis via diverse signaling pathways contributing to the regeneration of intervertebral disc.

Several *in vitro* studies at low pH levels demonstrated that the frequencies of proteoglycan synthesis in IVD were reduced significantly [40, 42–44]. Furthermore, studies demonstrated that IVD-like pH triggered a substantial reduction in cell viability, cell proliferation, and aggrecan secretion of AD-MSCs, which means that pH was an essential factor inhibiting biological and metabolic potency of AD-MSCs [40, 42–44]. Also, the secretion of collagen type-I was not altered or marginally elevated under IVD-like pH, though the acidic pH triggered cell viability as well as proliferation (Figure 2) [17]. Moreover, the secretion of collagen type-I in AD-MSCs was not different when grafted into the degenerative IVD under a low pH [17]. Another study revealed that pH was an essential factor, restricting the use of MSCs for disc repair because in most cases, the pH in a sternly degenerated disc is usually as low as 5.7 [45].

Studies have demonstrated that substantial MSCs reactions at a lower pH were observed in moderately or severely IVDD, especially considering the log nature of the pH scale [17, 45]. Nevertheless, when MSCs were subjected to pH values as low as 6.5 in a follow-up study, stern influence on gene expression, proliferation, and viability was observed [46]. Studies have also shown that bovine NPCs reduced the synthesis of sulfated GAG below pH 6.8 (Figure 2) [42, 44]. Bibby and Urban found a reduction in cell viability at pH to 6.7 and a clearer reduction at pH 6.2, particularly, when culturing cells under nutrient deficit microenvironments [47]. Therefore, ECM acidity was fundamental for MSC gene secretion and proliferation and may also speedup IVDD by negatively influencing resident cells [47]. Moreover, ASIC3 secretion by IVD cells was capable of facilitating IVD cells to adapt more easily to an acidic microenvironment compared to undifferentiated MSCs (Figure 2).

## 5. Osmolarity

Osmotic changes are evident in the physicochemical microenvironment with IVD cells or NPCs, as changes in disc mechanical burden resulted in substantial fluctuations in tissue hydration. Thus, osmotic pressure has a significant influence on the IVD cells' milieu [48, 49]. Mavrogonatou and Kletsas showed that high osmolarity reduced the proliferation frequency of NP IVD cells without loss of viability by a value of 500 mOsm/kg H_2_O, as well as their capability of novel DNA synthesis in a reversible manner [48]. They explained that the obvious blockade consequence could be due to a G_2_/M interruption taking place early after the introduction of high salinity complemented with a reduced S-phase population and G1 arrest [48].

The ECM of annulus in the physiological microenvironment contains aggrecan, which is the major type of proteoglycan and gylcosaminoglycan, which are negatively charged [40, 50]. These negative charges explain the high osmotic pressure of the IVD [40, 50]. It was established that compressing the IVD resulted in modifications in its hydration and osmolarity. The osmotic pressure in the NP ranges from 450–550 mOsm [51]. Mavrogonatou and Kletsas observed that augmented osmolarity triggered p38 MAPK and ATM-p53-p21WAF1-pRb pathway in IVD cells (Figure 2), which stemmed the cells in G2 and G1 phases of the cell cycle [48].

Mavrogonatou and Kletsas demonstrated that proliferation reduced considerably under the IVD-like high osmolarity [48]. They indicated that high osmolarity was capable of activating a p53-dependent DNA repair reactions such as phosphorylation and collection of histones H2A.X at the IVDD site [48]. Liang et al. observed that high osmolarity demonstrated to be an essential nutrient for the survival of IVD cells after AD-MSCs grafting [40]. They indicated that IVD-like high osmolarity substantially decreased the viability and proliferation of AD-MSCs [40]. They further stated that high osmolarity severely blocked the synthesis of aggrecan and collagen type-I (Figure 2) [40]. Nevertheless, initial studies demonstrated that hyperosmolarity had no substantial influence on proteoglycan content but upmodulated collagen type-I secretion in NP and AF cells [52].

Another study revealed that the quantity of proteoglycans was absolutely associated with the osmolality of ECM in IVD and the loss of proteoglycans resulted in the reduction of osmotic pressure during IVDD [53]. Furthermore, high osmolarity reduced the secretion of proteins that preserve the function of NP [53]. Tao et al. found a reduction of SRY-related HMG box (SOX)-9, aggrecan secretion in NPCs, downregulation of collagen type-II, and aggrecan secretion in NP-MSCs in high osmolarity (Figure 2) [54]. Their data suggest that IVD-like high osmolarity stimulated IVDD via the blockade of cell proliferation and ECM synthesis [54]. Similar studies observed the reduction in the number and viability of the IVD cells, particularly, NPCs together with loss of the IVD ECM as the triggers of IVDD [55, 56]. Further studies are needed in this direction to rectify conflicting finding on the effect of osmolarity on MSCs at the IVD microenvironment.

## 6. Glucose

Glucose levels are very crucial in adenosine triphosphate (ATP) production and ECM protein synthesis in the IVD because the cellular energy metabolism is predominant via anaerobic glycolysis [47, 57, 58]. Discrepancies in nutrient supply often result in the development of IVDD-associated pathological features such as cartilage endplate calcification and reduction in glucose concentrations [57]. A study revealed that glucose was capable of triggering senescence and diminishing osteogenic differentiation potential in classical plastic-adherent MSC [59]. Studies have shown that high glucose media or elevated plasma glucose concentrations trigger senescence and reduce proliferation frequencies and augmented apoptosis in rat MSCs [59–61].

Currently, media composed of 5.5 mM glucose are recommended and most often used for human MSCs in culture [62]. It is worth noting that serum glucose concentration in normal human beings is 5.55 mM [60, 63]. An augmented proliferation was observed in 25 mM glucose compared to 5 mM glucose in a study involving human umbilical cord blood-derived passaged MSC [64, 65]. Furthermore, SOX-2 secretary levels were augmented in 25 mM glucose, while octamer-binding transcription factor (OCT)-4 and FOXO3a secretory levels were not changed. Also, glucose triggered the TGF-*β* section via the protein kinase C (PKC) phosphorylation, resulting in the augmented proliferation of MSC (Figure 2) [60, 66]. Moreover, high glucose was capable of inhibiting growth factor initiation in rat multipotent adult progenitor cells, which are almost like MSCs [63].

Al-Qarakhli et al. demonstrated that MSCs under hyperglycemia exhibited insignificant NANOG, OCT-4 pluripotency, and CD34/CD45 hematopoietic markers (Figure 2) [67]. Liu et al. also demonstrated that NP-derived MSCs cultured in high glucose exhibited negligible secretion of stemness genes, mRNA, as well as protein secretion of silent information regulator protein 1 (SIRT1), SIRT6, glucose transporter 1(GLUT1), and HIF-1*α*, but augmented cell senescence, cell apoptosis, and caspase-3 secretion (Figure 2) [68].

Cheng et al. demonstrated that both adipose-derived stem cells (ADSCs) from diabetic donors (dADSCs) and nondiabetic donors (nADSCs) showed possibilities of transdifferentiation into neuron-like cells via a reactive oxygen species (ROS)-mediated mechanism when cultured in a suitable stimulation medium [1]. They further indicated that dADSCs or high-glucose-preserved nADSCs exhibited higher secretion of the pluripotent markers SOX-2, OCT-4, and nanog homeobox (NANOG) (Figure 2), whose effects were associated with ROS-mediated Akt inhibition [1].

Cheng et al. concluded that hyperglycemia has a slight influence on the identification markers but reduces the stemness of MSCs in most instances [1]. Markers for identification and stemness are key subjects of importance within the field of MSC senescence under hyperglycemic microenvironment [69]. Cheng et al. further established that the secretion of cell surface markers in dADSCs and nADSCs was analogous to that of MSCs [1]. Liang et al. demonstrated that IVD-like low glucose in the *in vivo* microenvironments may be a positive factor for AD-MSCs transplantation because it conserved cell viability as well as proliferation at comparatively normal levels and enriched the secretion of ECM proteins [40]. Further studies are needed in this direction to rectify conflicting finding on the effect of glucose on MSCs at the IVD microenvironment.

## 7. Hypoxia

Hypoxia is often referred to low O_2_ tension at the microenvironment of cells [70, 71]. The hypoxic microenvironment was capable of supporting the multipotentiality of a subpopulation of human bone marrow stromal cells (BMSCs) throughout osteogenic differentiation [70, 71]. Studies have demonstrated that human BMSCs boosted proliferative activity under the hypoxic microenvironment of 1.5–3% O_2_, compared to normoxia [71, 72]. It was further established that hypoxia enriched BMSC chondrogenic differentiation potential compared to normoxia [70, 71].

Also, there was an upsurge in proliferation rate compared to cells expansion under normoxia when ovine BMSCs were isolated and propagated under the hypoxia microenvironment of 5% O_2_ [70]. Furthermore, it was observed that ovine BMSCs isolated and expanded in hypoxic microenvironments, and successive chondrogenic differentiation in normoxia microenvironments showed an augmented chondrogenic phenotype relative to their counterparts after normoxia-intermediated isolation and expansion [70, 73].

Mueller et al. demonstrated that, under hypoxic milieu, the expansion culture of human BMSCs triggered chondrogenesis even under normoxic milieu [74]. Adesida et al. demonstrated that the BMSCs obtained via propagation, under low O_2_ tension, underwent a more vigorous chondrogenesis than their counterparts under normal O_2_ tension, albeit with dependence on the donor [70]. The robust chondrogenic capabilities were depicted with higher GAG per DNA content, enhanced transcript secretion of a group of chondrogenic genes such as aggrecan, collagen type-X alpha 1 chain (Col2a1). and SOX-9 and an extreme as well as even dissemination of collagen type-II and safranin O staining for sulfated proteoglycans (Figure 2) [70].

Studies have shown that transcriptional actions of SOX-9 augmented the gene promoter behaviors of aggrecan in chondrocytes [75, 76]. Furthermore, studies have demonstrated that transcriptional factors such as SOX-9, L-SOX-5, and SOX-6 are very crucial for the regulation of the secretion of Col2a1 and other genes associated with chondrogenesis (Figure 2) [75, 77]. Also, enhancement of chondrogenic potential under hypoxia was complemented with a simultaneous inhibition of Col10a1 secretion and a marker of hypertrophic chondrogenesis and a terminal differentiation of chondrocytes [70]. Hirao et al. demonstrated that, under hypoxia of 5% O_2_, enhanced chondrogenic differentiation of pluripotent MSCs line-like C3HT10T1/2 rather than osteogenesis via the machinery associated with simultaneous reduction of Col10a1 and runt-related transcription factor (RUNX)-2 activity through SMAD6 inhibition and histone deactylase 4 activation (HDAC4) (Figure 2) [78].

Adesida et al. indicate that the reaction of BMSCs to the hypoxic milieu is associated with the augmentation of transcriptional secretion of HIF-2*α* rather than HIF-1*α*, which remained uninterrupted [70]. Also, HIF-1*α* was involved in the hypoxia-mediated blockade of senescence and maintenance of human MSC properties [79]. It was further established that the reaction of the BMSCs to hypoxia was mediated by the transcriptional activity of HIF-2*α* [70]. Moreover, in the hypoxic milieu of 5% O_2_, HIF-2*α* triggered the secretion of chondrogenic genes such as Col2a1, aggrecan, and SOX-9 during chondrogenesis of AD-MSCs (Figure 2) [80].

Studies have shown that BM-MSCs and ADSCs are capable of augmenting cell viability and synthesis of ECM components in the hypoxic microenvironment of 1–5% O_2_ and low glucose microenvironment *in vitro* [4, 17, 40]. Studies have further demonstrated that BM-MSCs had a greater capability of developing colony-forming units, reduced osteogenic differentiation, and inhibited IL-1*β* (Figure 2) chondrogenic differentiation in the hypoxic microenvironment [81, 82]. Also, MSC senescence and conserved cell stemness via stimulation of telomerase activity through the HIF1*α*-twist-mediated downregulation of the E2A-p21 pathway (Figure 2) were inhibited in hypoxic microenvironment [4, 79].

These adaptive modalities above may enhance MSC survival in the hostile IVD milieu after grafting. Also, persistent exposure to stern hypoxia with O_2_ <1% together with serum deprivation led to total cell death [4, 83]. Hypoxia together with TGF-*β*1 (Figure 2) simultaneously triggered gene cassettes within the NPCs that encode for ECM and cell surface receptors. Furthermore, low O_2_ tension together with TGF-*β*1 activated ERK and p38 signaling pathways (Figure 2), resulting in eventual differentiation of MSC [4, 84].

Risbud et al. established that in the presence of hypoxia and TGF-*β*1, cultured MSCs secreted a phenotype coherent with NPCs via mitogen-activated protein kinase (MAPK) signaling pathways. Hypoxia triggered an upsurge of MM-2, collagen type-II and IX, and aggrecan secretion, while TGF-*β*1 treatment augmented collagen type-II and aggrecan gene secretion (Figure 2) [13].

## 8. Apoptosis

IVD cells undergo programmed cell death through one of three apoptosis pathways such as mitochondrial permeabilization of the outer membrane, death receptor, and endoplasmic reticulum pathways [3]. The maintenance of the IVD often depends on the integrity of the ECM [3]. It is well established that degenerative activities within the ECM microenvironment trigger an upsurge in catabolism, which internally triggers several biological modifications in the IVD cells, resulting in the stimulation of the different apoptosis pathways [3]. The various IVD cell apoptosis pathways are usually stimulated at different levels of the IVDD [3].

It was established that the mild stage of IVDD usually involves the endoplasmic reticulum pathway. Also, the mild/moderate stage of IVDD was usually characterized by the death receptor pathway, while the severe stage of IVDD was characterized by the mitochondrial pathway [3]. The stimulation of bcl-2 and blockade of caspase-3 are the parameters for measuring reduction in apoptosis, while higher HIF-1*α* and CXCR4 mRNA secretion are the parameters for measuring improvement in cell migration [85, 86].

Liu et al. established that human NP-MSCs exhibited a decrease in cell proliferation, augmented apoptosis, and decreased gene secretion of type-I and II collagen, aggrecan, and SOX-9, as well as of stemness-associated genes such as OCT-4, NANOG, JAGGED, and NOTCH1 in an *in vitro* study at a decreased pH of 7.4–6 (Figure 2) [36]. It was further established that NPCy underwent irreversible apoptosis via caspase-dependent mitochondrial pathways. Moreover, a specific kind of programmed cell death with necrosis-like qualities (necroptosis) was implicated in the compression-stimulated death of NPCy [4, 87].

It was also observed that the presence of AD-MSCs triggered a decrease in NPCy apoptosis via the blockade of caspase-3 and 9 as well as augmentation of ECM genes with decreased secretion of metalloproteinases and proinflammatory cytokines like IL-1*β*, IL-6, and TNF*α* in a coculture experiment involving AD-MSCs and NPCy at a prolonged compressive loading of 3 MPa for 48 h (Figure 2) [4, 88]. Although Wangler et al. did not indicate any higher fraction of apoptotic IVD cells following MSC migration [85], an upsurge in apoptotic cells was seen in bovine IVDs at day 5 of culture compared to newly (day 0) isolated IVDs, which could be due to associated nonphysiological *ex vivo* culture and potential nutrient deprivation under free swelling microenvironment [89].

Wangler et al. established a minimal but inconsequential decrease in apoptotic cells after MSC treatment, signifying a marginal effect of MSCs on IVD cell apoptosis [85]. They demonstrated that MSC homing augmented a proportion of tyrosine kinase endothelial receptor (TEK)-positive IVD cells in bovine as well as human IVD samples [85]. They further revealed that a proliferative reaction, as well as lower proportion of dead cells, was seen after MSC homing in both bovine as well as human IVD tissues [85]. Nevertheless, a positive correlation was detected between the upregulation of TEK as well as B-cell lymphoma 2 (Bcl2) transcripts (Figure 2) [86]. It was established that Bcl2 was observed as an antagonist of the apoptosis cascade because it counteracted the apoptosis-promoting factor Bax resulting in an upregulation of Bcl2 secretion, thus contributing to an enhanced survival of phenotypic IVD cells upon MSC homing [86].

## 9. Pyroptosis

Pyroptosis is another kind of programmed cell death, which is activated by inflammasomes like NOD-, LRR-, and pyrin domain-containing proteins (NLRP) and absent in melanoma 2 (AIM2)-like receptor proteins and tripartite motif-containing proteins [90]. Studies have shown that the cleavage of inflammatory mediators such as caspase-1 or caspase-11 results in the stimulation of cells to express proinflammatory cytokines such as IL-1*β* and IL-18 once these inflammasomes are activated [90, 91]. Zhang et al. established an IVDD model by IVD puncture and discovered that NLRP3-mediated NP cell pyroptosis was triggered in the succession of IVDD, with the activation of the NLRP-3 inflammasome and the elevation of caspase-1, cleaved gasdermin D (GSDMD), and augmented expression of cytokines IL-18 and IL-1*β* [90].

He et al. demonstrated that NLRP3-mediated NP cell pyroptosis was involved in the IVDD progression both *in vivo* and *in vitro* under the propionibacterium acnes activation [92]. Tang et al. also demonstrated that NLRP3 inflammasome was stimulated in NPCs after H_2_O_2_ activation [93]. Song et al. detected stimulation of NLRP-3 in patients. They indicated that dysregulation of NLRP3 was responsible for the development of IVDD [94]. Tavakoli Dargani et al. observed that doxorubicin exposure appreciably triggered the secretion of NLRP-3, which, in turn, stimulated pyroptosis in H9c2 cells [95, 96]. They indicated further that pyroptosis was inhibitable with the treatment of exosomes derived from embryonic stem cells [95]. They further established that exosome treatment exhibited an analogous effect *in vivo*, which was advantageous in ameliorating doxorubicin-stimulated cardiomyopathy [96].

Studies have shown that lipopolysaccharide (LPS) was capable of triggering an inflammatory response and facilitating proinflammatory cytokines buildup in NP cells [90, 97]. Lebeaupin et al. established that LPS was capable of stimulating NLRP3-induced pyroptosis *in vivo* [98]. Thus, NP cell pyroptosis is very crucial in the formation of IVDD. Zhang et al. detected that MSCs were capable of inhibiting NP cell pyroptosis blocking the secretion of NLRP3 in the LPS-induced model [90]. They indicated further that the anti-pyroptosis effect of MSCs was abrogated when they treated the MSCs with GW4869 to inhibit the secretion of exosomes (Figure 2) [90]. They concluded that the effect of MSCs on pyroptosis was mainly caused by its derived exosomes [90]. Zhang et al. ,therefore, determined novel machinery via which the downregulation of NLRP3 by exosomal miR-410 led to a reduction in caspase-1 and GSDMD, thereby blocking NP cell pyroptosis to ameliorate IVDD (Figure 2) [90].

## 10. Hydrogel

Hydrogels are the common biomaterial scaffolds for tissue engineering purposes due to their hydrated nature [99, 100]. Most hydrogels have the capability of mimicking the natural structure of *in vivo* tissue milieus [99, 100]. Hydrogels possess properties such as biocompatibility, a flexible method of formation, estimated physical features, offering of structural integrity to tissue building, fundamental structural, and compositional resemblances to ECM and a widespread framework, which offers cellular proliferation and survival [99, 100]. These properties make them the ultimate tissues for cell delivery and transient support for NP tissue regeneration [99, 100].

The amalgamation of human MSC at low O_2_ tension as well as mimicking the three-dimensional (3-D) ECM milieu via hydrogels was capable of augmenting the differentiation capabilities of human MSC towards an NP cell variety [99, 100]. Kumar et al. established that novel synthetic p(HEMA-co-APMA) gPAA hydrogel was able to encapsulate human MSC *in situ* and enhance the growth and differentiation of cartilage-like cells [99]. They observed a significant reduction in stiffness of NP tissue and detected modulus when human MSCs were cultured in human MSC or chondrogenic media after being encapsulated in hydrogels [99]. The reason for such occurrence was as a result of polymer degradation transpiring via hydrolysis [99].

Photocurable hydrogel together with hypoxic and chondrogenic culture media supported differentiation of human MSC towards a chondrocyte-like lineage and augment secretory levels of markers such as collagen II and aggrecan [99, 101]. Thus, by mimicking the structural milieu as well as the hydrated state of NP tissue using the hydrogel and by providing TGF-*β*1 in chondrogenic media together with a hypoxic milieu led to transcriptional changes in human MSC which resulted in an augmented secretion of aggrecan and collagen II leading to outside-in and inside-out signaling between the cells and their milieu, which ultimately resulted in the expression as well as production of NP-like tissue ECM [99, 101].

## 11. Conclusion

IVD microenvironment clues such as nucleopulpocytes, pH, osmotic changes, glucose, hypoxia, apoptosis, pyroptosis, and hydrogels are capable of influencing the MSCs during the treatment of IVDD. Therefore, clinical usage of MSCs ought to take into consideration these microenvironment clues during treatment. These microenvironment clues could function as prognostic indicators during the treatment of patients with IVDD using MSCs.

## Data Availability

No data were used to support this paper.

## Abbreviations

AF: Annulus fibrosus
ASICs: Acid-sensing ion channels
ADAMTS: A disintegrin and metalloproteinase with thrombospondin motifs
AQP2: Aquaporin-2
AD-MSC: Adipose-derived mesenchymal stem cells
ADSCs: Adipose-derived stem cells
ATP: Adenosine triphosphate
AIM2: Absent in melanoma 2
bFGF: Basic fibroblast-like growth factor
BDNF: Brain-derived neurotrophic factor
BMP: Bone morphogenetic protein
BGT: Betaine/*γ*-aminobutyric acid transporter
BMSCs: Bone marrow stromal cells
Bcl2: B-cell lymphoma 2
CEPs: Cartilaginous endplates
CCL: C-C motif chemokine ligand
Col2a1: Collagen type-X alpha 1 chain
ECM: Extracellular membrane/matrix
ERK: Extracellular signal-related kinases
Gli: Glioma-associated oncogene
GPC3: Glypican 3
GDF: Growth differentiation factor
GLUT1: Glucose transporter 1
GSDMD: Gasdermin D
HIF: Hypoxia-inducible factor
HSP: Heat shock protein
HDAC4: Histone deactylase 4 activation
IVD: Intervertebral disc
IVDD: Intervertebral disc degeneration
IGF-1: Insulin-like growth factor 1
IL: Interleukin
KRT: Keratin
LPS: Lipopolysaccharide
MSCs: Mesenchymal stem cells
MMP: Matrix metalloproteinases
MAPK: Mitogen-activated protein kinase
NP: Nucleus pulposus
NPCs: Nucleus pulposus cells
NPCys: Nucleopulpocytes
NGF: Nerve growth factor
NOS-2: Nitric oxide synthase 2
NP-MSCs: Nucleus pulposus mesenchymal stem cells
NANOG: Nanog homeobox
NLRP: NOD-, LRR-, and pyrin domain-containing proteins
OCT: Octamer-binding transcription factor
pH: Potential of hydrogen
PDGF: Platelet-derived growth factor
PKC: Protein kinase C
SMIT: Sodium myo-inositol transporter
SOX: SRY-related HMG box
SIRT1: Silent information regulator protein 1
ROS: Reactive oxygen species
RUNX: Runt-related transcription factor
TGF-*β*: Transforming growth factor beta
TonEBP: Tonicity enhancer-binding protein
TonE: Tonicity-responsive enhancer element
TauT: Taurine transporter
TEK: Tyrosine kinase endothelial receptor
3-D: Three-dimensional.
